# Coincidental SARS-CoV-2 infection and mRNA vaccination: a case report addressing the most important clinical questions

**DOI:** 10.1007/s15010-021-01613-w

**Published:** 2021-05-07

**Authors:** Ozan E. Eren, Matthias Tonon, Florian Schöberl, Clemens Gießen-Jung, Andreas Moosmann, Alexandra Hollaus, Maximilian Muenchhoff, Konstantinos Dimitriadis

**Affiliations:** 1 Department of Neurology, University Hospital, LMU Munich, Marchioninistr. 15, 81377 Munich, Germany; 2 Max Von Pettenkofer Institute, LMU Munich, Munich, Germany; 3 German Center for Infection Research, Munich Partner Site, Munich, Germany; 4 COVID-19 Registry of the LMU Munich (CORKUM), University Hospital, LMU Munich, Munich, Germany; 5 Department of Medicine III, University Hospital, LMU Munich, Munich, Germany; 6 Institute for Stroke and Dementia Research (ISD), University Hospital, LMU Munich, Munich, Germany

**Keywords:** Vaccination, COVID-19, Infection, Antibodies, Coincidental

## Abstract

The case describes the coincidental mRNA vaccination and SARS-CoV-2 infection of a 31-year-old physician addressing the theoretical considerations and recommendations for further actions in such a particular constellation that we will expect more often in the near future.

## Introduction

Severe acute respiratory syndrome coronavirus 2 (SARS-CoV-2) infection resulting in what the world has known as the COVID-19 pandemic has been dominating our scientific as well as private lives since the past year [1]. Thankfully, desperately needed vaccines of different working mechanisms have been proven effective in clinical trials lately. Therefore, in Germany, the first legally approved vaccine named Comirnaty (BNT162b2, Pfizer–BioNTech) made the start off with vaccinating in late December 2020. Besides prevention of SARS-CoV-2 infection by this vaccine, there is also a new mechanism of action due to the development of mRNA-based vaccines [2]. The immune response to a SARS-CoV-2 infection is not understood in all details, but what we know so far is, that the median time to seroconversion for both IgG and IgM is around 2 weeks after onset of symptoms [3, 4]. However, antibody responses do not inevitably develop at the same time, quality and quantity in all patients, instead substantially depending on the severity of the disease course with significantly higher peaks in critically affected patients [3, 4]. Another interesting observation in SARS-CoV-2 infection, as also in other viruses, is that the theoretically expected time sequence of first IgM and then IgG being detectable is not always reality, with IgG succeeding over or appearing at the same time as IgM [3]. The impact to the sequence of antibody appearance in the lately infected patient is not described. Additionally, most COVID-19 patients developed at least one antigen specific antibody to a SARS-CoV-2 infection, including anti-viral, anti-spike, and anti-n protein IgM or IgG [5]. As for the vaccination with BNT162b2, encoding full-length spike, mainly S-binding IgG, would be expected to be detected, besides the additionally described raised specific CD4+ and CD8+ T-cell responses [5–7].

The following presented case is unique due to coincidental mRNA vaccination and SARS-CoV-2 infection addressing the theoretical considerations and recommendations for further actions in such a particular constellation.

## Case description

Here, we present the case of a 31-year-old male physician working on a specialized non-intensive-care COVID-19 infection unit developing flu-like symptoms a few hours after the first application of the Pfizer–BioNTech COVID-19 vaccine (Comirnaty, BNT162b2) starting with headache, generalized limb pain and coughing as well as chills and increased temperature in the following night. After the symptoms did not improve during the following day, a nasopharyngeal swab was acquired and tested for a SARS-CoV-2 infection which carried out to be positive with 32 million copies/ml. Due to the lack of respiratory symptoms, the colleague was sent into home isolation.

Due to the coincidence of the first-time use of the Pfizer–BioNTech mRNA vaccine with flu-like symptoms with highly positive SARS-CoV-2 copies in the nasopharyngeal swab, the same four questions were raised by different clinicians. 1. How can we be sure that the symptoms were not only side effects of the vaccination and in fact an infection with SARS-CoV-2? 2. Could the mRNA vaccine cause a false positive PCR result? 3. If the vaccination was at the same time of a coincidental infection with SARS-CoV-2, would it be safe? 4. Would there be a difference in the immune response in such a special constellation?

To answer our questions on day 7 (T1) after the symptom onset, i.e., 6 days after the first PCR test, we tested for SARS-CoV-2 antibody responses using commercially available serological assays and for T-cell responses using an enzyme-linked-immuno-spot-assay (ELISPOT assay) [for further study, see 8]. A nasopharyngeal swab was taken showing a decrease in copies to 1.7 million copies/ml. We repeated the same procedure again 10 days later (T2), by then showing a further decrease in viral load to 0.013 million copies/ml as well as a seroconversion for the nucleocapsid (N) IgG (Abbott 7.54 index; mixed IgG/IgM/IgA Roche Cobas e411 34.67 COI) and spike (S) protein IgG (Abbott, 216 AU/ml), implicating that at that point, there was only a seroconversion regarding IgG but not IgM (Abbott 0.55 AU/ml) (see Table 1). The ELISPOT assay being zero before showed now for SARS-CoV-2-binding proteins 27 spots for S1, 12 spots for S2, and 28 spots for *N* per 250.000 cells (see Table 1).

**Table 1:** Changes in viral load, Ig response, and ELISPOT assay

	T1	T2	T3
Roche Ig total nucleocapsid > 1 pos		0.128*	34.76
Abbott IgG nucleocapsid Index > 1,4 pos		0.07*	7.54
Abbott IgM spike Index > 1 pos		0.06*	0.55
SARS CoV-2 viral load in 10^6^ copies/ml	32	1.7	0.013
ELISPOT SARS-CoV-2 S1 per 250,000 cells		0*	27
ELISPOT SARS-CoV-2 S2 per 250,000 cells		0*	12
ELISPOT SARS-CoV-2 N per 250,000 cells		0*	28

T1 = day 1 after symptom onset = first PCR, T2 = day 7 after symptom onset = first sample antibody and ELISPOT, T3 = day 17 after symptom onset = second sample antibody and ELISPOT assay

^*^Below cut-off

## Discussion

Until now, experiences with mRNA vaccines in a real-world setting are limited. Regarding its pandemic character and an increasing number of vaccinations with mRNA vaccines against SARS-CoV-2 in the early future, more such cases like the described one will be expected. Therefore, with this unique case, we want to critically discuss the theoretical considerations, which guided our further action plan. Our experiences and findings should help to generate hypothesis for larger observational trials in such an extraordinary scenario.

First, new onset of symptoms particularly in highly exposed target populations such as health care workers must be suspicious of a coincidental infection with SARS-CoV-2 and cannot be ignored as possible vaccination side effect, therefore prompting testing and appropriate safety precautions.

Second, one should not be concerned of the PCR test becoming false positive through the vaccination itself. It is highly unlikely that mRNA administrated by vaccination into the deltoid muscle could be detected in the nasopharyngeal mucosa. Even if traces of vaccine-introduced RNA would be taken up by a swab, only those components of diagnostic PCR-based assays that target the spike-gene could theoretically become positive. Since most FDA- and CE-certified assays rely on at least two different targets, this would be identified.

Third, in our case, the vaccination seemed to be well tolerated despite an existing infection with SARS-CoV-2 not showing an overregulation of the immune system. Blood results (including also but not only inflammation values like CRP, Il-6, procalcitonin, leukocytes; liver and kidney values) did not show any pathological lab results 7 days after symptom onset and vaccination.

Fourth, at the first timepoint 7 days post-immunization, no SARS-CoV-2-specific antibodies were detected. On day 17 after vaccination, seroconversion had occurred showing IgG responses against SARS-CoV-2 nucleocapsid and spike protein. In case of immunity induced by the vaccine alone, only the induction of Ig against the spike protein but not nucleocapsid would occur, as the latter one is not expressed by the mRNA of the vaccine [6, 9]. Therefore, antibody responses against the nucleocapsid protein can be used to distinguish humoral immunity in vaccines compared to natural infection. Additionally, the ELISPOT assay showed comparable T-cell responses against the nucleocapsid and spike peptide pools.

On a side note, since there is no recommendation so far how to proceed in cases of coincidental infection at the same time of vaccination and what the minimum interval should be [10], we administrated the second vaccination following the commonly recommended vaccination schedule 21 days later. The second injection was well tolerated only causing mild side effects (i.e., mild headache, pain in the limbs and sleeplessness). Further research has to answer the optimal duration between the vaccination and the time to wait for “boosting” a past infection.

## Conclusion

Due to the incredible progress made in the last few weeks and the start of universal vaccination, the case described here will be a more often scenario, so that an algorithm for recommended actions in such a situation gains weight. Based on our experiences, we can draw the following concrete conclusions:

1. There is no general need for SARS-CoV-2 testing before vaccination, since simultaneous infection and vaccination seem not to increase the risk for a more severe course of SARS-CoV-2 infection (i.e., hospitalization and need of oxygen supply).
2. Our observations do not justify additional (prophylactic) therapies (e.g., steroids) beyond general recommendations.
3. We do not know whether a second vaccine dose is obligatory in such a scenario and if, when it should be applied. Further studies are needed to address this issue. At least in our case, the usual time schedule of 21 days was well tolerated.
4. Finally, we recommend rapid testing for an additional possible SARS-COV-2 infection in case of alleged vaccination reactions lasting longer than 1 day.

Our observations in this special constellation should trigger the development of hypothesis for future observational and/or randomized-controlled clinical trials.
